# A Study on the Expression of BCR-ABL Transcript in Mixed Phenotype Acute Leukemia (MPAL) Cases Using the Reverse Transcriptase Polymerase Reaction Assay (RT-PCR) and its Correlation with Hematological Remission Status Post Initial Induction Therapy

**DOI:** 10.4084/MJHID.2012.024

**Published:** 2012-05-08

**Authors:** Prateek Bhatia, Jogeshwar Binota, Neelam Varma, Deepak Bansal, Amita Trehan, Ram Kumar Marwaha, Pankaj Malhotra, Subhash Varma

**Affiliations:** 1 Assistant Professor-Pediatrics; 2 Senior Laboratory Technician; 3 Professor and Head -Hematology; 4 Additional Professor; 5 Professor – Pediatric Hemato-oncology and; 5 Additional Professor; 6 Professor & Head – Internal Medicine, Post Graduate Institute of Medical Education and Research, Chandigarh

## Abstract

**Introduction:**

The MPAL comprise 2–5% of all acute leukemia. The present WHO 2008 classification has separated two groups in MPAL based on t(9;22) positivity and MLL rearrangement.

**Aims & Objectives:**

The aim of the present pilot study is to note the frequency of BCR-ABL transcript in MPAL cases using the RT-PCR assay and to correlate the status with hematological remission post induction.

**Materials & Methods:**

A total of 10 MPAL cases classified on Flow-cytometry based on the current WHO 2008 criteria were enrolled. In all the cases Bone marrow or peripheral blood sample in EDTA was processed for molecular studies and the RT-PCR reaction carried out using primers specific to the t (9;22) and t(4;11) translocation. The post induction check marrow slides were also reviewed.

**Results:**

Out of the total 10 MPAL cases, 7/10 (70%) were adult and 3/10 (30%) pediatric cases. A total of 4/10 (40%) cases showed positivity for the t(9;22) transcript and none for t (4;11). Of the 4 positive cases, 3/10(30%) were adult cases and 1/10(10%) pediatric case. The BCR-ABL transcript type in adult cases was b3a2 (p210) in 2/3 (66%) and e1a2 (p190) in 1/3 (33.3%) case. The single pediatric case was positive for b3a2 transcript.

**Discussion & Conclusion:**

All the 4 positive MPAL cases presented with high TLC and low platelet count (p<0.05). The positive cases also showed hematological remission at post induction check marrow (blasts<5%). This could partly be explained due to good response to the imatinib added to the treatment protocol.

## Introduction

Acute leukemia with a mixed phenotype is a rare disease and comprises 2–5% of all acute leukemias. Immunophenotyping for B/T/Myeloid markers is necessary for detection of Mixed Phenotype acute leukemias. The WHO 2008 classification identifies two subtypes of MPAL with characteristic genetic lesion as separate entities:- i) MPAL with t (9;22) and ii) MPAL with MLL rearrangements. The incidence of MPAL with t(9;22) and MLL rearrangement in adults is 28–30% and 2–3% and in children 3–5% and 10–15% respectively^1^. The aim of the present study was to note the frequency of BCR-ABL positivity in MPAL cases using the Reverse Transcriptase Polymerase Reaction Assay (RT-PCR) and to correlate BCR-ABL positivity with status of hematological remission at 1^st^ check marrow.

## Materials and Methods

The study was a prospective study carried out over a period of one year from July 2010-June 2011. Inclusion criteria included: (i) All cases of Acute leukemia coming for Bone marrow examination to Department of Hematology, PGIMER, Chandigarh and diagnosed as Mixed Phenotype Acute Leukemia according to WHO 2008 criteria on Bone marrow examination and Immunophenotyping. (ii) All MPAL Cases receiving treatment in the Institute and having complete follow-up details of 1^st^ check marrow. Cases of Acute leukemia with aberrant B/T/Myeloid marker expression on immunophenotyping, known cases of Chronic Myeloid leukemia (CML) in Blast crisis and cases with incomplete follow-up details/not receiving treatment in the institute were excluded from the study.

## Methodology

RNA was extracted from 1–2ml Bone Marrow aspirate in EDTA or 3–5ml Peripheral blood (if blasts > 20% and TLC> 50X10^9^ /L) using the commercial kit (Qiagen Miniamp RNA Blood kit) according to manufacturer’s instructions. The RNA quality was checked in each case by absorbance at 260nm in a spectrophotometer and by running a 1% formaldehyde gel for 18s and 28s RNA bands. This was followed by cDNA synthesis according to the commercial kit protocol (Fermantas cDNA kit). The quality of cDNA was checked using primers for β-Actin housekeeping gene. Reverse Transcriptase polymerase chain reaction was carried out using primers specific for p210 (b3a2 and b2a2)^2^ adapted from the study of Jones et al and the protocol was standardized in our laboratory in 2004 for detection of p210 transcript in CML cases. A separate Multiplex RT-PCR was done for detection of p190 and MLL-AF4 transcript as adapted from study by Pakakasama et al^3^. This Multiplex RT-PCR is routinely used in our laboratory as a screening PCR for detection of common chimeric fusion transcripts in ALL cases. The PCR conditions for both the PCR are outlined below and the primer sequences and the product base size are highlighted in the **table 1**. The BCR-ABL PCR for p210 transcripts (b3a2 and b2a2) was carried out in following conditions: Pre-Denaturation- 94c-4 mts –one cycle; Denaturation-94c-1 mt, Annealing-63c-2 mt and Extension- 72c- 3 mt for 32 cycles. This was followed by one cycle of final Extension at 72c-10 mt. The Multiplex RT-PCR for p190 (e1a2) transcript and the MLL-AF4 transcript was carried out as follows: The PCR was carried out in a final volume of 25 ul with 1 ul cDNA, 1x PCR buffer, 200 uM dNTP, 1.5 mM MgCl2, 120 nM of each primer pair and 1 unit of Tag polymerase. After initial denaturation step at 94° C for 3 min, the 35 cycles of PCR condition including 94° C for 45 s, 63° C for 1 min, and 72° C for 1.5 min were performed. The final extension for 7 min at 72° C was set to ensure a complete extension of all PCR products. The PCR products were then run on agarose gel and stained with Ethidium Bromide and visualized under UV-Gel doc for b3a2 (385bp), b2a2 (310bp) and e1a2 (521bp) bands. Appropriate positive and negative controls were included in each run.

Review of Geimsa stained Bone marrow and peripheral blood slides at 1^st^ check marrow (Day 14-Pediatric cases & Day 28-Adult cases) for Remission status (Standard Remission criteria- Hb>10g/dl; TLC 4–12X 10^9^/L; Platelet 150–450 × 10^9^/L; Bone marrow blasts<5%; No Auer Rod; No blasts in peripheral blood) was performed and relevant data analyzed. Cytogenetic samples were taken in all cases.

## Ethical Justification

The blood sample used in the study is withdrawn as a part of routine diagnostic work-up of the patient and no additional sample pricks were performed. Prior informed consent was taken from all patients/guardians before withdrawl of sample.

## Statistical Analysis

The X^2^ and the Fischer exact test were used to correlate the clinical and laboratory features in the two groups and a p value of ≤0.05 was taken as significant.

## Results

The total frequency of MPAL was 6% (10/170) of all acute leukemia cases that underwent routine diagnostic evaluation during the study period. Out of the 10 MPAL cases, 7/10 (70%) were adult and 3/10 (30%) were Pediatric cases. The age in adult cases ranged from 26–60 years (Mean age 31.5 yrs) and M: F ratio was 1.3:1. The age in Pediatric cases ranged from 7 months – 7 years (Mean age 3.7 years) and all were Male children. The clinical and Hematological data of the MPAL cases is outlined in **table 2**.

On Immunophenotyping, 60% (6/10) were B/Myeloid and 40% (4/10) were T/Myeloid. The incidence of BCR-ABL positivity in MPAL cases was 40% (4/10). All four MPAL cases positive for BCR-ABL transcript presented with High TLC and Low platelet count (p value ≤0.05). 3/4 (75%) BCR-ABL positive MPAL’s had B/Myeloid phenotype while 1/4 (25%) had T/Myeloid phenotype. Molecular breakpoint was p210 (b3a2 type) in 3/4 (75%) cases and p190 in 1/4 (25%) case. The Immunophenotyping, molecular and remission data in MPAL cases is detailed in **table 3**. Cytogenetics revealed satisfactory metaphases in 6/10 cases. Philadelphia positive metaphases were noted in all four BCR-ABL positive MPAL cases with a 100% correlation. However, no additional cytogenetic abnormality could be identified in any of the cases.

**Figure 1.**
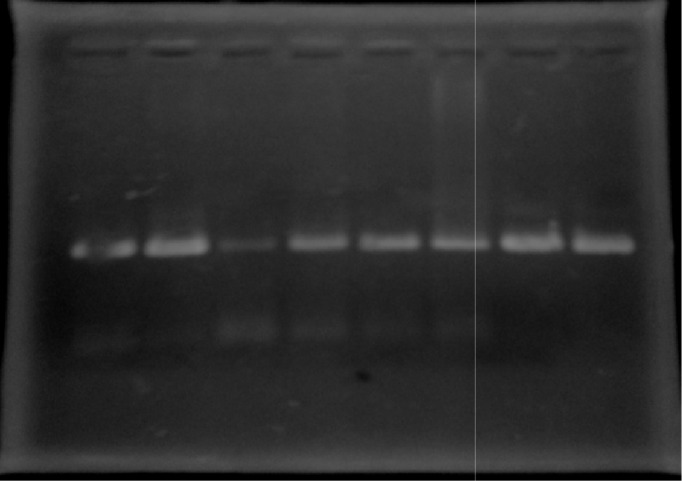
Beta Actin band positivity as marker of internal cDNA quality in all 4 positive MPAL cases (Two per case)

**Figure 2.**
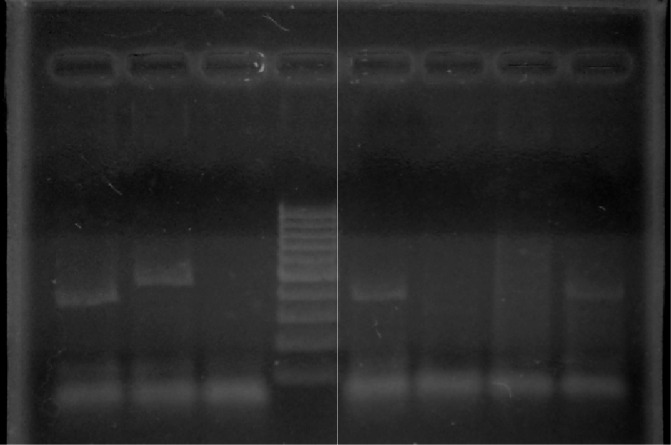
Agarose gel showing BCR-ABL positivity in the four MPAL cases. Lane 1: Case 1 (Positive; b3a2 transcript- 385bp). Lane 2: Case 6 (Positive; e1a2 transcript- 521bp). Lane 4: Ladder pattern 100bp. Lane 5: Case 2 (Positive; b3a2 transcript- 385bp). Lane 8: Case 10 (Positive; b3a2 transcript- 385bp).

**Figure 1 and 2** show RT-PCR agarose gels for Housekeeping gene (β-actin) and BCR-ABL transcript positive MPAL cases. None of the MPAL cases showed positivity for MLL-AF4 transcript.

## Discussion

**Figure 3.**
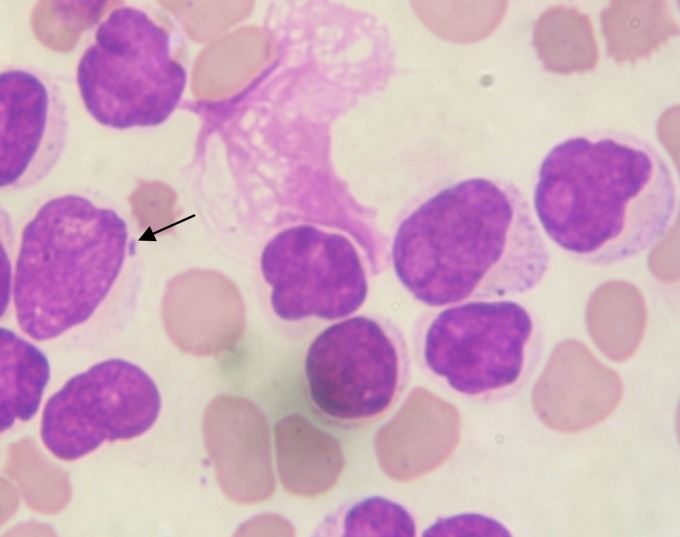
Geimsa Stain- PBF (1000X); Two population of Blasts. Arrow- Blast showing presence of Auer Rod like granules

In the present study all the MPAL cases positive for BCR-ABL transcript had high WBC count and low platelet count (p<0.05). Many studies have also shown BCR-ABL positive MPAL’s presenting with high WBC count^4^,^5^, but present study also shows relation of BCR-ABL positivity with low platelet count. There were 4 cases of T/Myeloid MPAL,of which one showed BCR-ABL positivity (**Figure 3**). Mastutes et al^6^ in their study had also found BCR-ABL positivity in 13% (2/15) cases with T/Myeloid phenotype.

The incidence of MPAL cases was 6% and the BCR-ABL positivity in present study was 40% which is quiet comparable to few other studies from the subcontinent and west. Studies from Asian subcontinent by Xu et al^7^, Lee et al^8^ and Mi et al^9^ found incidence of MPAL as 4.6%, 2.1% and 3.4% and BCR-ABL positivity as 25.0%, 36.8% and 16.7% respectively. Studies from west by Owaidah et al^10^, Carbonell et al^11^, Legrand et al^12^, Killick et al^13^ and Weir et al^14^ found the incidence of MPAL as 3.4%, 4.0%, 8.0%, 3.6% and 1.3% and BCR-ABL positivity as 9.1%, 30.8%, 35.0%, 38.1% and 18.8% respectively.

Check marrow is performed at Day 14 in Pediatric Acute leukemia cases and Day 28 in adult cases. The Complete Hematological Remission rate (CHR) at first check marrow after induction therapy was 50% (5/10) in MPAL cases; 30% cases had no follow-up details while 20% were not in CHR. Of the four BCR-ABL positive cases 75% (3/4) were in CHR and all these were positive for b3a2 transcript type (p<0.05%). Only one BCR-ABL positive MPAL (p190 transcript) showed 60% blasts in Bone marrow at 1^st^ check marrow. However, the case number and positivity for p190 transcript is low and the follow-up data is limited to derive any conclusive statement regarding prognostic difference between the two transcript types in MPAL cases. The MPAL cases are treated with ALL/AML standard induction protocol based on predominant Blast immunophenotype. In addition Imatinib is added to the treatment regimen if the MPAL case is BCR-ABL positive on RT-PCR. All the above four BCR-ABL positive MPAL cases received Imatinib along with standard induction regimen drugs. The good response in three cases could be attributed to the possible favorable effect of Imatinib addition to the standard induction regimen, which is quiet well described in literature. However long term follow up data in more number of cases is needed to further substantiate the findings. None of our MPAL case was positive for MLL-AF4 transcript. However we had not looked for other MLL rearrangements in these cases.

## Conclusions

Immunophenotyping of all acute leukemia cases is necessary with a complete panel of lineage specific markers to detect presence of Mixed Phenotype Acute leukemias. RT-PCR for BCR-ABL is also mandatory in all MPAL cases as the overall prognosis in these patients may improve with addition of Imatinib (Gleevac /STI571) therapy to the treatment regimen.

## Figures and Tables

**Table 1.** Primer sequences and band sizes for BCR-ABL and MLL-AF4 transcript

**S. No.**	**Transcript**	**Primer sequence**	**Product size (bp)**
1.	b3a2 (p210)	BCR-b2 5′-ACAGAATTCCGCTGACCATCAATAAG-3′ ABL-a2 5′-TGTTGACTGGCGTGATGTAGTTGCTTGG-3′	385
2.	b2a2 (p210)	BCR-b2 5′-ACAGAATTCCGCTGACCATCAATAAG-3′ ABL-a2 5′-TGTTGACTGGCGTGATGTAGTTGCTTGG-3′	310
3.	e1a2 (p190)	BCR-e1 5′-GAC TGC AGC TCC AAT GAG AAC-3′ ABL-a2 5′-GTT TGG GCT TCA CAC CAT TCC-3′	521
4.	MLL-AF4	MLL 5′-CCG CCT CAG CCA CCT AC-3′ AF4 5′-TGT CAC TGA GCT GAA GGT CG-3′	559

**Table 2.** Clinical presentation and Hematological data in MPAL cases

**S.NO.**	**AGE (YRS)**	**SEX**	**Lymph Node Enlargement**	**Hepatomegaly**	**spleenomegaly**	**WBC COUNT (× 10^9^/L)**	**PLATELET COUNT (x 10^9^/L)**
CASE 1	3.5	M	+^*^ (Inguinal)	+ (Mild)	+ (Mild)	19.2	45
CASE 2	42	F	-	+ (Mild)	+ (Mild)	202.4	30
CASE 3	26	F	++^**^(cervical)	-	-	3.1	243
CASE 4	7	M	-	-	-	33.4	31
CASE 5	31	M	-	+(Mild)	+ (Mild)	51	25
CASE 6	15	M	+ (Cervical)	-	-	72	11
CASE 7	27	F	+++(cervical, axillary and Inguinal)	-	-	6.5	252
CASE 8	28	M	-	-	-	5.1	381
CASE 9	60	F	-	+(Mild)	+ (Moderate)	246.6	53
CASE 10	7/12	M	-	-	-	97.4	118

**Table 3.** Immunophenotype and Molecular data and Hematological remission status in MPAL cases.

**S.NO.**	**IMMUNOPHENOTYPIC PROFILE**		**MOLECULAR PROFILE**	**Post Induction check marrow (Hematological Remission Status)**
	**(B/MYELOID)**	**(T/MYELOID)**	**BCR-ABL (9;22)**	
CASE 1	CD 19 bright,10,20 & CD 13, 33, 117, Anti-MPO		POSITIVE-p210 (b3a2) Transcript	In Remission
CASE 2		CD 2, 3, 4, 7, cCD3, CD 56 (>90%) and CD 13, 117, Anti-MPO (> 30%)	POSITIVE-p210 (b3a2) Transcript	In Remission
CASE 3		cCD3, 2, 5, 7, 4, 8, TdT and CD13, 33, Anti-MPO	-	Not in Remission (9% blasts)
CASE 4		cCD3, 2, 5, 7, TdT, 4, 8, TCR dim and CD13, 33, Anti-MPO	-	No follow-up
CASE 5	CD 19 bright, 10 (>97%) and Anti-MPO (>70% Blasts)		-	In Remission
CASE 6	CD 19 bright, CD 10 and CD 13, 33, Anti-MPO, 11b, 11c, 4dim, 34, 123, 45		POSITIVE-p190 (e1a2) Transcript	Not in Remission (60% blasts)
CASE 7		cCD3, 2, 5, 7, 4, 8, TdT and CD13, Anti-MPO	-	No follow-up
CASE 8	CD 19, 22, 79a, 34, DR, 123, 38, Tdt and CD 13, 33, 117, Anti-MPO		-	No follow-up
CASE 9	CD 19, 34, DR, 123, 38, Tdt, and CD 13, 33, 117, Anti-MPO		POSITIVE-p210 (b3a2) Transcript	In Remission
CASE 10	CD 19, 22, 79a (>40%) and CD 4, 14, 16, 33, DR, Anti-MPO (> 60% Blasts)- B/Monocytoid- Infantile Leukemia		-	In Remission
**TOTAL 10**	**6/10 (60%)**	**4/10 (40%)**	**4/10 (40%)**	
